# Development of a behavioural modulation strategy for disease control based on network interventions

**DOI:** 10.1098/rsos.251322

**Published:** 2025-11-26

**Authors:** Hanqi Zhang, Zhongkui Sun, Nannan Zhao, Yuanyuan Liu, Shutong Liu

**Affiliations:** ^1^ Northwestern Polytechnical University, Xi'an, Shaanxi, People’s Republic of China; ^2^ Chang'an University, Xi'an, Shaanxi, People’s Republic of China; ^3^ Xi'an University of Posts and Telecommunications, Xi'an, Shaanxi, People’s Republic of China; ^4^ Shaanxi Normal University, Xi'an, Shaanxi, People’s Republic of China

**Keywords:** behavioural epidemiology, microscopic Markov chain, network intervention, evolutionary game, multiplex networks

## Abstract

The impact of human behaviour evolution poses a major challenge in the control of COVID-19. The key to overcoming this problem is incorporating behavioural factors into disease interventions. This paper proposes a novel behavioural modulation means based on network intervention strategies, aiming to achieve disease prevention at the population level through behavioural modulation of seed nodes. Taking individual decision-making behaviour as a representative example, we explore the efficacy of the proposed behavioural modulation method within a coupled behaviour-disease model. Using epidemic threshold and infection density as indicators, the results demonstrate that behavioural modulation under various network intervention strategies can effectively control disease transmission within populations. On this basis, the intervention costs incurred by implementing behavioural modulation are also noteworthy. Further analysis reveals an optimal interval of intervention proportions capable of simultaneously achieving epidemic control and cost savings, which can guide the practical implementation of such control measures. The above conclusions are validated through simulation with representative real-world contact network data. Our work has led to new advances in realizing disease control from a behavioural perspective, which is of great value as a guide for the public health sector in the development of epidemic prevention policies.

## 1. Introduction

During COVID-19, a wide variety of disease interventions have rapidly emerged globally. Scholars have made efforts ranging from vaccination reminders [1], contact tracing [2] and web-based application development [3] to curb the disease’s spread. In this process, network interventions are drawing great attention due to their broad reach and sustainability [4]. Indeed, the influence of social networks has been revealed as an important determinant of disease prevalence patterns [5,6], and network interventions represent a process of using social networks to accelerate behavioural change or improve organizational performance [7]. The network intervention strategies proposed from individual identification, segmentation, induction and alteration perspectives provide fruitful results for COVID-19 prevention [8–10]. Despite the developments, recent research has also exposed the dilemma that network interventions can raise, which is that they may instead cause an increase in the basic reproduction number when changes in contact behaviour are not uniform [11]. Similar problems are also evident when applied to obesity interventions; that is, the network intervention strategies fail to achieve the results expected by the designers [12,13]. This gap is rooted in the fact that human behaviour is dynamic, and ignoring the influence of behavioural factors will lead to incomplete capture of disease transmission characteristics. Inspired by this, we aim to propose a novel approach to suppress disease spread within populations by implementing behavioural modulation on seed nodes with high influence that are identified through network interventions.

Changes in individual behaviour during an epidemic are often motivated by real-time perceptions of the infection risk and represent decisions made after weighing costs and benefits [14]. Recent research into the decision-making behaviour dynamics of individual non-pharmacological interventions shows the potential for complex phenomena such as periodic oscillations [15,16], social dilemmas [17] and behavioural compensations [18] to occur in the system. The above findings demonstrate that individual decision-making behaviour exerts a significant influence on disease transmission processes. Consequently, we select the decision-making process as the core mechanism for developing novel behavioural modulation methods. Much like the spread of viruses, decision-making behaviours can also diffuse among individuals through information communication. People would always tend to imitate the strategies adopted by other individuals who achieve greater benefits. Such interactions of individual strategies within complex network environments are typically described by the framework of evolutionary game theory [19–21]. Building upon the foregoing theoretical basis, we further design the following details of the modulation method. When the decision-making behaviour of seed nodes undergoes directed modulation, owing to their central influence within the population, the strategies they adopt will rapidly propagate to other individuals, thereby driving the renewal of group decisions. This strategy of blocking disease spread by modulating the decision-making behaviour of key nodes will be a new advancement in the application field of network interventions.

As a further step, to validate the practical efficacy of the proposed behavioural modulation strategy in disease control, it is necessary to establish a suitable model as a foundation. Behavioural drivers are critical to infectious disease dynamics, and disease spread can in turn result in the renewal of human behaviour, thus constituting a coupled disease–behaviour system [22–25]. To integrate information from multiple aspects of a coupled system, the multilayer network is developed as a generally applicable framework [26,27]. Of these, the multiplex network that shares the same set of nodes in multiple layers is the most compatible with coupled disease–behaviour studies, facilitating the analysis of features and interactions of the same group of people in terms of both disease and behaviour [28,29]. We therefore choose a two-layer multiplex network as the basic structure of the dynamic model. The upper layer represents the behaviour layer, which portrays the evolution of decision-making behaviour, while the lower layer is the contact layer that describes the health state of individuals. The variation rules for the probability of an individual being in different states over time are expressed by the microscopic Markov chain approach (MMCA) [30]. This approach analyses the state transfer probabilities from a discrete time viewpoint and is often used in multiplex network models [31–33]. Aligning our proposed modulation strategy with the coupled dynamics model will allow for a more intuitive demonstration of the implementation effects.

The remainder of the paper is arranged as follows. In §2, we give a behaviour–epidemic model considering the evolutionary game framework by the MMCA method, and the periodic oscillations induced by behavioural factors are shown in §3. The behavioural modulation means are introduced in §4, and the optimal intervention proportion interval for controlling disease is determined. The above conclusions are validated by simulation results under real-world networks in §5. Finally, §6 summarizes the main findings and outlooks of future research directions.

## 2. The game–epidemic dynamics model

As the basis for implementing behavioural modulation, our proposed model integrates individual decision-making behaviour with disease transmission. On the one hand, individuals’ strategic choices are characterized through an evolutionary game framework. Each individual is provided with two epidemic coping strategies, support (T) and disregard (D) the government responses. During pandemic events, government departments usually introduce control policies to call on people to enhance self-protection. At this point, groups adopting the T strategy are able to reduce the likelihood of being infected by implementing protection, while, on the contrary, those adopting the D strategy will remain at a higher risk of getting the disease. Noteworthy is that individuals are allowed to update their strategies based on pay-offs at each discrete moment. We choose the Fermi function from evolutionary game theory [34,35] to denote an individual’s strategy update probability as


(2.1) $$\Gamma \left(\pi_{i},\pi_{j}\right)=\frac{1}{1+e^{-\left(\pi_{j}-\pi_{i}\right)/\omega }},$$

where $\pi_{i}$ and $\pi_{j}$ are the pay-offs of individual i and j, respectively, ω is the selection behaviour intensity and $\Gamma (\pi_{i},\pi_{j})$ represents the probability that individual i chooses to imitate the strategy chosen by individual j. It is not difficult to find that the higher the pay-off of individual j, the more likely individual i will choose to imitate j to update his/her strategy. This real-time strategy updating is more in line with the actual features of human behaviour.

On the other hand, the disease transmission is simulated by the classical susceptible–infected–susceptible (SIS) model. Each individual in an infected state can infect other susceptible individuals with probability β, as well as regain health and re-enter the susceptible state with probability μ. As mentioned earlier, it is assumed that individuals who adopt a strategy supporting the government responses will thus reduce their own infection risk, which is reflected in a reduced probability of being infected as dβ (0<d<1). We express the meaning of the parameter d as the government response strength, where strict regulation will promote the decay of the infection rate. Up to this point, it can be concluded that there are four states that an individual may exhibit in the game-disease model, namely, susceptible disregarding government response (DS), infected disregarding government response (DI), susceptible supporting government response (TS) and infected supporting government response (TI). Among them, supporting government control requires greater effort from the individual, while being infected is hazardous to the individual’s health. We therefore denote the cost to be paid in these two states by the constants $C_{T}$ and $C_{I}$ and assume that $C_{I}>C_{T}$ to ensure the rationality of the strategy selection, and the pay-off corresponding to each of the four states can be expressed as


(2.2) $$\begin{array}{lcr} & \begin{array}{r} \pi^{DS}=0,\pi^{DI}=-C_{I},\pi^{TS}=-C_{T},\pi^{TI}=-C_{I}-C_{T}. \end{array} & \end{array}$$

On this basis, the total cost that individual i needs to pay is the weighted sum of all the states denoted as $\pi_{i}\left(t\right)=\sum_{C}\pi_{i}^{C}p_{i}^{C}\left(t\right),C\in \left\{DS,DI,TS,TI\right\}$ according to the probability of being in different states. In this way, individual heterogeneity is ensured to be reflected in the pay-off.

**Figure 1.**
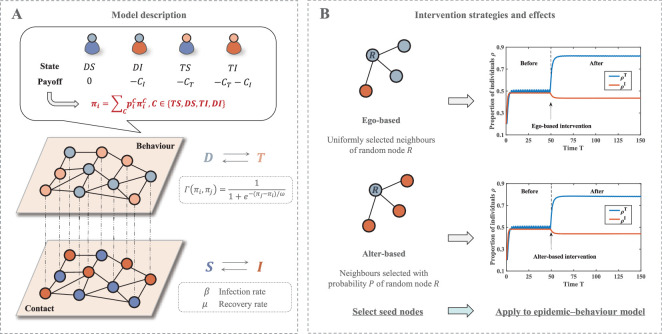
Schematic overview of the main work in this research. (A) Schematic of the game–epidemic model on multiplex networks. There are four possible states that an individual may exhibit, each corresponding to a different pay-off. The total pay-off of an individual is the weighted sum over different states. The behaviour and contact layers have the same network connecting edge structure, and each follows different state transition rules. (B) Two network intervention strategies are applied to select seed nodes. The disease dynamics are then affected by modulating the decision-making behaviour of the seed nodes. The implementation of interventions promotes support for the government and reduces the infection level.

A key issue is that both the imitative behaviour of strategies and the spread of disease require inter-individual connectivity pathways as a foundation. In the two-layer multiplex network here, the upper layer is a behaviour layer, where individuals with connected edges will learn about each other’s pay-offs and complete decision-making behaviours, and the lower layer is a contact layer, where the disease will spread from the infected to the susceptible through contacts between individuals. The feature of multiplex networks sharing the same set of nodes in multiple layers [36] allows us to treat each node as an individual and to simultaneously understand the performance of a population in terms of both behaviour and disease. In general, the spread of epidemics relies on face-to-face contact with virus carriers. During such interactions, individuals may also communicate their strategic choices and apply the received information to update their own decision-making. Thus, within the context where the network serves to describe face-to-face contact patterns among individuals, both layers are assumed to adopt an identical network structure. Here, we give a schematic of the game-disease model on the multiplex network in figure 1, followed by the derivation of model expressions and theoretical analyses.

### 2.1. Model equations

**Figure 2.**
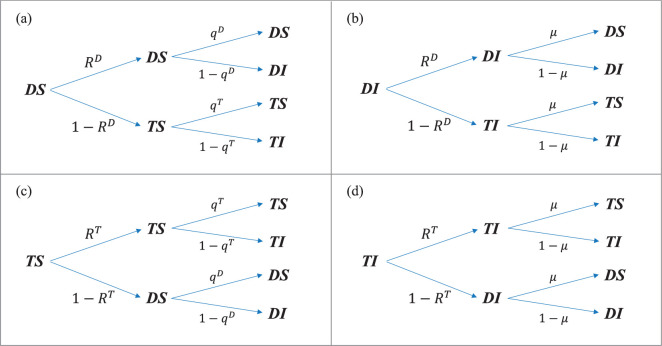
The probability tree describing the state transition paths of an individual with respect to both behaviour and health. Following the multiplex network structure, the state transfer of an individual is distinguished into two phases, starting with the upper behaviour layer and then the lower contact layer. The probability of each state update occurring is labelled on the arrows.

The master equations of the coupled system are constructed in a discrete time frame, obtained by means of the MMCA. In a population of size N, the probability for individual i (1≤i≤N) to be in state C∈{DS,DI,TS,TI} at time t is specified as $p_{i}^{C}\left(t\right)$. For conceptual distinction, we denote the adjacency matrices of the behavioural and contact layers as $A={\left\{a_{ij}\right\}}_{N\times N}$ and $B={\left\{b_{ij}\right\}}_{N\times N}$, respectively. According to the previous description, they are supposed to be equal. To capture the state transition characteristics in the behaviour layer, we denote the probability that an individual i who adopts a strategy of disregarding (D) or supporting (T) the government responses at time t chooses to keep his/her current strategy selection unchanged as


(2.3) $$\begin{array}{r} R_{i}^{D}(t)=\prod_{j=1}^{N}\left(1-a_{ji}p_{j}^{T}(t)\Gamma \left(\pi_{i}\left(t\right),\pi_{j}\left(t\right)\right)\right),\\ R_{i}^{T}(t)=\prod_{j=1}^{N}\left(1-a_{ji}p_{j}^{D}(t)\Gamma \left(\pi_{i}\left(t\right),\pi_{j}\left(t\right)\right)\right), \end{array}$$

where $p_{i}^{D}\left(t\right)=p_{i}^{DS}\left(t\right)+p_{i}^{DI}\left(t\right),$ $\;p_{i}^{T}\left(t\right)=p_{i}^{TS}\left(t\right)+p_{i}^{TI}\left(t\right)$. Further, for the disease transmission state, the probability that a D-adopter or a T-adopter i remains avoiding infection and in a susceptible state at time t is assumed to be


(2.4) $$q_{i}^{D}(t)=\prod_{j=1}^{N}\left(1-b_{ji}p_{j}^{I}(t)\beta \right),\;q_{i}^{T}(t)=\prod_{j=1}^{N}\left(1-b_{ji}p_{j}^{I}(t)d\beta \right),$$

respectively, where $p_{i}^{I}\left(t\right)=p_{i}^{DI}\left(t\right)+p_{i}^{TI}\left(t\right)$. Given the numerous variables and parameters involved in this system, for ease of understanding, all symbols and their definitions are summarized in table 1 of appendix A. To clearly represent the paths and probabilities of state transitions, the transition probability trees are given in figure 2.

On the basis of the state transfer probability tree, we can derive the possible state update outcome at the next moment in a discrete time frame. The methodology for constructing transition probability trees and the detailed derivation of MMCA equations are presented in appendix B. The main equation of the game-epidemiological dynamics model can be concluded as


(2.5) $$\begin{array}{rl} p_{i}^{DS}(t+1) & =p_{i}^{DS}(t)R_{i}^{D}(t)q_{i}^{D}(t)+p_{i}^{DI}(t)R_{i}^{D}(t)\mu +p_{i}^{TS}(t)\left(1-R_{i}^{T}(t)\right)q_{i}^{D}(t)+p_{i}^{TI}(t)\left(1-R_{i}^{T}(t)\right)\mu ,\\ p_{i}^{DI}(t+1) & =p_{i}^{DS}(t)R_{i}^{D}(t)\left(1-q_{i}^{D}(t)\right)+p_{i}^{DI}(t)R_{i}^{D}(t)\left(1-\mu \right)+p_{i}^{TS}(t)\left(1-R_{i}^{T}(t)\right)\left(1-q_{i}^{D}(t)\right)\\ & +p_{i}^{TI}(t)\left(1-R_{i}^{T}(t)\right)\left(1-\mu \right),\\ p_{i}^{TS}(t+1) & =p_{i}^{DS}(t)\left(1-R_{i}^{D}(t)\right)q_{i}^{T}(t)+p_{i}^{DI}(t)\left(1-R_{i}^{D}(t)\right)\mu +p_{i}^{TS}(t)R_{i}^{T}(t)q_{i}^{T}(t)+p_{i}^{TI}(t)R_{i}^{T}(t)\mu ,\\ p_{i}^{TI}(t+1) & =p_{i}^{DS}(t)\left(1-R_{i}^{D}(t)\right)\left(1-q_{i}^{T}(t)\right)+p_{i}^{DI}(t)\left(1-R_{i}^{D}(t)\right)\left(1-\mu \right)\\ & +p_{i}^{TS}(t)R_{i}^{T}(t)\left(1-q_{i}^{T}(t)\right)+p_{i}^{TI}(t)R_{i}^{T}(t)\left(1-\mu \right). \end{array}$$

### 2.2. Epidemic threshold

Theoretical analysis of the epidemic threshold is one of the key steps in epidemic dynamics studies and can serve as a powerful guide to quickly assess the steady state of disease. For system (2.5), we perform theoretical derivations and obtain the following theorem.

**Theorem 1.** *For the game–epidemic system* (2.5) *on multiplex networks, the epidemic threshold can be expressed as*


(2.6) $$\beta_{c}=\frac{\mu }{\Lambda_{\max}\left(H\right)},\;H={\left\{h_{ij}\right\}}_{N\times N}:h_{ji}=\left(1-\left(1-d\right)p_{i}^{T}\right)b_{ji},$$

*where* $\Lambda_{\max}\left(H\right)$ *denotes the largest eigenvalue of the matrix* H *and* $p_{i}^{T}=p_{i}^{TS}+p_{i}^{TI}$ *corresponds to a steady state. For the epidemic threshold* $\beta_{c}$*, it ensures that the disease-free equilibrium is locally stable when* $\beta <\beta_{c}$ *and unstable when* $\beta >\beta_{c}$.

*Proof.* Under the discrete time frame, the disease spread will gradually reach a steady state as time moves forward to t→∞. At this point, the probability of an individual being in different states will also show a stabilizing trend, which can be noted as $p_{i}^{DS}\left(t+1\right)=p_{i}^{DS}\left(t\right)=p_{i}^{DS},$ $\;p_{i}^{DI}\left(t+1\right)=p_{i}^{DI}\left(t\right)=p_{i}^{DI},$ $\;p_{i}^{TS}\left(t+1\right)=p_{i}^{TS}\left(t\right)=p_{i}^{TS},$ $\;p_{i}^{TI}\left(t+1\right)=p_{i}^{TI}\left(t\right)=p_{i}^{TI}.$Imagine that when the infection rate β is very close to the threshold $\beta_{c}$, i.e. $\beta \to \beta_{c}$, the infected density in the stationary solution must only take on very small values, which can then be denoted as $p_{i}^{I}=\varepsilon_{i}\ll 1$. Under this assumption, the probability expression in equation (2.4) can be approximated in the following form:


(2.7) $$\begin{array}{lcr} & \begin{array}{rl} q_{i}^{D}(t) & =\prod_{j=1}^{N}\left(1-b_{ji}p_{j}^{I}(t)\beta \right)=\prod_{j=1}^{N}\left(1-b_{ji}\varepsilon_{j}\beta \right)\approx 1-\beta \sum_{j}b_{ji}\varepsilon_{j},\\ q_{i}^{T}(t) & =\prod_{j=1}^{N}\left(1-b_{ji}p_{j}^{I}(t)d\beta \right)=\prod_{j=1}^{N}\left(1-b_{ji}\varepsilon_{j}d\beta \right)\approx 1-d\beta \sum_{j}b_{ji}\varepsilon_{j}. \end{array} & \end{array}$$

Substituting this equation into equation (2.5) and performing an omission on the higher-order terms gives


(2.8) $$\begin{array}{lcr} & \begin{array}{rl} p_{i}^{DS} & =p_{i}^{DS}R_{i}^{D}+p_{i}^{TS}\left(1-R_{i}^{T}\right),\\ p_{i}^{TS} & =p_{i}^{DS}\left(1-R_{i}^{D}\right)+p_{i}^{TS}R_{i}^{T},\\ \mu \varepsilon_{i} & =\left(p_{i}^{DS}\beta +p_{i}^{TS}d\beta \right)\sum_{j}b_{ji}\varepsilon_{j}. \end{array} & \end{array}$$

Further approximations can be applied to these equations. from $p_{i}^{I}=p_{i}^{DI}+p_{i}^{TI}$, we can deduce that $p_{i}^{DI}\ll 1$ and $p_{i}^{TI}\ll 1$, so that $p_{i}^{T}=p_{i}^{TS}+p_{i}^{TI}\approx p_{i}^{TS}$ and $p_{i}^{D}=p_{i}^{DS}+p_{i}^{DI}\approx p_{i}^{DS}$ hold, and therefore we get


(2.9) $$\begin{array}{lcr} & \sum_{j}\left\{\left[1-\left(1-d\right)p_{i}^{T}\right]b_{ji}-\frac{\mu }{\beta }\delta_{ji}\right\}\varepsilon_{j}=0, & \end{array}$$

where $\delta_{ji}$ stand for the elements in an identity matrix. This equation can be transformed into a matrix form as $\left(H-\frac{\mu }{\beta }E\right)\vec{\varepsilon }=\vec{0}$, in which the infected density vector $\vec{\varepsilon }={\left(\varepsilon_{1},\varepsilon_{2},\ldots ,\varepsilon_{N}\right)}^{T}$, and the matrix $H={\left\{h_{ij}\right\}}_{N\times N}$ satisfies $h_{ji}=\left(1-\left(1-d\right)p_{i}^{T}\right)b_{ji}$. If the disease enters a persistent outbreak, it implies that there are non-trivial solutions to this equation, thus μ/β is an eigenvalue of the matrix H. This allows for the critical infection rate that guarantees the extinction of the disease, i.e. the epidemic threshold can be expressed as


(2.10) $$\begin{array}{lcr} & \beta_{c}=\frac{\mu }{\Lambda_{\max}\left(H\right)} & \end{array}$$

by the largest eigenvalue $\Lambda_{\max}\left(H\right)$ of the matrix H. It can be concluded that the disease will die out if $\beta <\beta_{c}$, otherwise, it will keep spreading in the population.∎

## 3. Impact of behavioural factors

**Figure 3.**
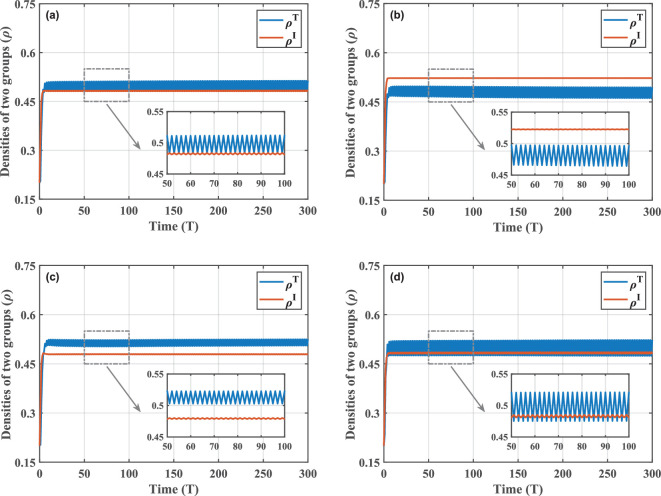
Time series of densities of T-adopters and infected individuals for various behavioural parameters. (a) Base case with parameter condition set as $N=500,C_{I}=2,C_{T}=0.2,\omega =0.5,d=0.6,\mu =0.5,\beta =0.3$. This figure serves as a comparison with the rest of the figures, which all change only one parameter compared with it. Parameters that have changed are (b) d=0.9, (c) $C_{I}=4$ and (d) ω=0.9. The smaller images embedded in the figures are localized enlargements. Under the influence of different behavioural factors, the coupled system exhibits diverse periodic features.

The MMCA theory provides a framework that allows us to better understand the crucial correlation between behaviour and disease from a discrete time perspective. In this context, time-course diagrams are more suitable for capturing the rich dynamics of behaviour. Therefore, we implement a series of numerical simulation efforts to demonstrate the impact that diverse behavioural performances may have on the epidemic situation. In the multiplex network, the same scale-free network is used for the behaviour and contact layers, built according to the idea of the Barabási–Albert model [37]. In addition, we select, to simultaneously observe variation trends in the densities of two groups, individuals who support government policies and those who are infected, to clarify the effects of the game–epidemic interaction, and they are denoted as $p_{i}^{T}\left(t\right)=p_{i}^{TS}\left(t\right)+p_{i}^{TI}\left(t\right)$ and $p_{i}^{I}\left(t\right)=p_{i}^{DI}\left(t\right)+p_{i}^{TI}\left(t\right)$. Schematic for the variation of $p_{i}^{T}\left(t\right)$ and $p_{i}^{I}\left(t\right)$ over time under different behavioural parameters is shown in figure 3. Of these, figure 3a is referred to as the base case and can be used for two-by-two comparisons with other figures to highlight the differences in results due to parameter variations.

According to numerical simulations, in strong contrast to the existing findings, the introduction of evolutionary game mechanisms allows disease transmission to exhibit stable periodicity characteristics. Moreover, this periodicity is consistently present under a wide range of behavioural parameters. For a clearer view of the periodic dynamics, we locally zoom in on the curves after entering the steady state in figure 3. It can be noticed that in each period of evolution, an increase in the number of T-adopters tends to be accompanied by a decrease in the density of infected individuals, and vice versa. The intuition behind this phenomenon lies in the feedback interplay between behaviour and disease. When more people begin to take enhanced precautions, the disease spread will be significantly hindered, which can be reflected as a reduction in the population infection level over the short term. This will be seen as a sign of decreased risk of getting infected. In turn, driven by the disparity of pay-offs, individuals will be inclined to forgo paying the extra prevention cost and instead adopt a strategy of disregarding government policy. This may provide an opportunity for the disease to develop in the gaps where group protection is relaxed, thus triggering a rebound in the infected density. It is such cyclic feedback that induces the periodic performance of the coupled system and demonstrates the bidirectional behaviour-disease effects intuitively.

With an understanding of the periodic dynamics caused by behavioural factors, we further analyse what effects the different decision-making behavioural performances have on the stable periodic state. Taking figure 3a as the base case, we first decrease the government response strength d and obtain the plot in (b). Since the parameter d indicates the decay level of the infection rate, the weakening of government responses will be reflected as an increase in the d value. The results reveal that the infection level is significantly higher when the government controls are less intense than in the base case, as the infection rate does not reach a sufficient attenuation. Inadequate effectiveness of protection also results in fewer individuals being supportive of government restrictions at the same time. As we have found in our previous studies [38], d is the behavioural parameter with the highest sensitivity level due to its ability to directly influence the epidemic situation through infection rates. Thus, we can detect significant changes in group densities at different d. For other behavioural parameters, they can only indirectly influence disease transmission by influencing decision-making behaviour. In (c) and (d), we examine the effects of cost parameters and selection behaviour intensity. Simulations indicate that the effect of their influences is more significant in the periodic oscillations of strategic choice behaviour. On the one hand, when the cost difference between the two strategies is even greater, individual decision-making becomes lopsided. The high cost of infection drives stronger public support for government policies. While $\rho^{T}$ increases, periodic fluctuations also weaken. On the other hand, an increase in the selection behaviour intensity promotes imitation of others’ strategies, and people will be more flexible in updating their strategies, increasing the oscillation amplitude. In an extreme scenario, it can be seen from the definition of the Fermi function that when ω→+∞, individual i may possess the right to randomly choose between two strategies independent of pay-offs. Conversely, when ω→0, individual i will unconditionally choose to imitate the strategy of j. All in all, the mutual influence between individual behaviour and disease spread is reflected in various aspects. If possible, regulation on individual behaviour can also be seen as one of the effective ways to control the epidemic course.

The above findings point to further directions for our research. Based on the game–epidemic model, is it possible to control diseases by modulating the decision-making behaviours of seed nodes selected by network intervention? Taking two network intervention strategies as examples, we conduct a preliminary experiment. The time-series plot in figure 1 displays a rapid decrease in the density of infected individuals and an increase in the proportion of individuals supporting the government within the system after exerting modulation. This demonstrates the feasibility of a strategy that combines network intervention with behavioural moderation. To obtain further results, we carry out detailed research work in the next chapter.

## 4. Analysis of intervention strategies

For the behaviour-disease model, we first introduce two intervention strategies to identify highly influential nodes (seed nodes) in the network. They are referred to as the ego-based strategy and the alter-based strategy, which are proposed by Kumar *et al.* based on their research about the friendship paradox. Both strategies determine seed nodes from the neighbours of a randomly selected node. The difference is that the ego-based strategy continues to randomly select a neighbour, whereas the alter-based one sets each neighbour to be selected with a probability P. The identical structure of the two-layer network ensures that the selected seed nodes exert significant influence in both behavioural diffusion and disease transmission. Details of the definition and implementation are described in [39]. In each simulation, the total number of seed nodes is set to K. We assume that the strategies of the selected seed nodes will be directly updated to support the government (T), and then we observe how implementing the behavioural modulation affects epidemic development.

### 4.1. Effectiveness of interventions

**Figure 4.**
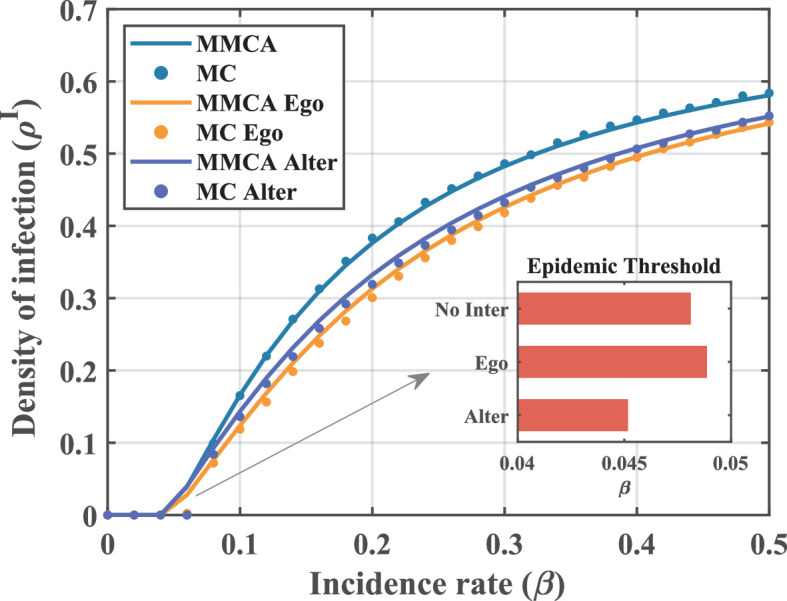
The variation curve of steady infection density $\rho^{I}$ with incidence rate β under different scenarios. Parameter conditions are set as $K=0.4*N,N=500,C_{I}=2,C_{T}=0.2,$ ω=0.5,d=0.8,μ=0.5,P=0.4. To avoid the effects of period oscillations and get steady solutions, the infection density is derived by averaging the results of the last 100 time steps. Solid lines represent the numerical results of MMCA models, and scatters stand for MC simulation results averaged over 50 independent experiments. The bar charts show the epidemic threshold results for different scenarios calculated according to theoretical analysis. Both intervention strategies are effective in reducing infection levels, with the ego-based one performing better.

Monte Carlo (MC) simulations are often used to validate the accuracy of models constructed by the MMCA method. We present the results of MMCA and MC simulations for two intervention and no-intervention scenarios in figure 4. Firstly, the consistency of the results under both MMCA and MC methods proves that our proposed model can be sufficiently accurate in describing the coupled dynamics features, which is the basis for the subsequent research to be carried out.

In figure 4, we can observe the impact of implementing interventions on the epidemic situation along two types of indicators, namely, infection density and epidemic thresholds. Compared with the no-intervention scenario, the reduction in the infected individual density is noticed after the introduction of two interventions, which suggests that modulation of decision-making behaviour in seed nodes is effective for disease prevention. Comparing the two strategies, lower densities of infected individuals and higher epidemic thresholds can be achieved under an ego-based intervention. In terms of control effects, it would be more applicable to disease transmission models under behavioural coupling. We believe the reason for this phenomenon lies in the indirect nature of alter-based strategies when selecting seed nodes, which may lead to intervention delays and thus declining efficiency. Moreover, according to the pioneering study [39], the relative efficiency of two strategies is also highly dependent on network characteristics. Within the contact network we constructed, edges are widely spread across nodes of differing degrees. This may induce the network to exhibit a positive inversity, thereby enabling the ego-based strategy to demonstrate better performance in reducing infection levels. Notably, we also find from the time-series plot in figure 1 that the imposition of interventions attenuates the periodic oscillations of the system. So at this point, an alter-based intervention would be more conducive to the stability of the epidemic situation. Taken together, the two intervention strategies are each advantageous in different ways, but both promote the outbreak situation in a direction that is favourable to the population.

### 4.2. Optimal intervention proportion

**Figure 5.**
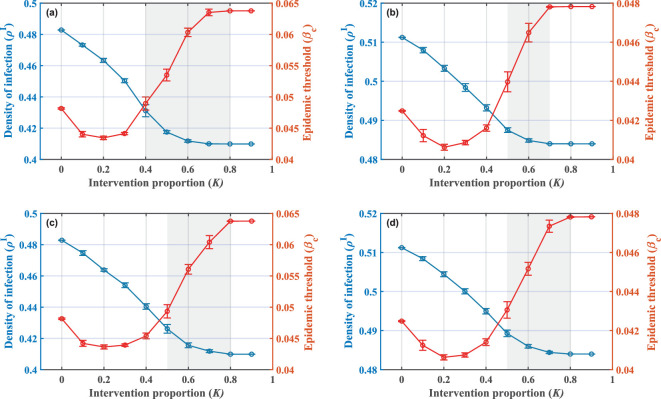
Double curve plot of infection density (left blue axis) and epidemic threshold (right red axis) as a function of the number of seed nodes. Parameter conditions are set as $N=500,C_{I}=2,C_{T}=0.2,\omega =0.5,\beta =0.3,\mu =0.5,P=0.4$ and (a) d=0.6, ego-based, (b) d=0.8, ego-based, (c) d=0.6, alter-based, (d) d=0.8, alter-based. K=0 represents the no-intervention scenario. Data points here represent the average of multiple simulations, with error bars based on the standard deviation. Simulations indicate the existence of a recommended optimal interval capable of ensuring both disease control and cost savings, as shown by the grey areas.

Although the current results have demonstrated the feasibility of behavioural modulations, the details of the intervention implementation need to be further explored. The larger the size of seed nodes, namely, the higher the intervention proportion, the higher the management cost to the government departments. In this context, a question that attracts our attention is whether there is an optimal intervention proportion that can effectively control disease spread while saving management resources to a maximum extent. When the infection density $\rho^{I}$ and the epidemic threshold $\beta_{c}$ are regarded as the judgement indicators, lower $\rho^{I}$ and higher $\beta_{c}$ indicate that the epidemic situation is under control. The curves of both with the intervention proportion are shown in figure 5.

On the one hand, in terms of the infection density, it is observed under both interventions that as the intervention proportion increases, the infection density reduces, and the effectiveness of prevention is improved. Notably, in the case of an extremely high intervention proportion, the density of infected individuals will no longer change with it, as will the epidemic threshold. We believe the intuition behind this phenomenon can be explained as follows. The initial point of network intervention strategies is to identify highly connected individuals in the population and to spread the control effect to the whole population by targeting these individuals on a small scale. A too-high intervention proportion is contrary to the original goal and can no longer have a positive impact on the prevention effects. Hence, we emphasize that blindly scaling up interventions is not an optimal response strategy during an epidemic. Not only does it fail to further enhance the effectiveness of prevention and control, but it also results in a waste of public resources.

On the other hand, for the epidemic threshold, we find that at a small intervention proportion, lower results are obtained under both interventions than in the no-intervention scenario. The reason for this phenomenon can be analysed theoretically from the expression of the epidemic threshold. The application of interventions will firstly promote the individual’s choice of a government supportive strategy, which will be manifested as a rise in $p_{i}^{T}$. Accordingly, the values of the elements of matrix H in equation (2.6) become smaller. This may trigger an increase in the spectral radius of matrix H, resulting in a decrease in the epidemic threshold at lower intervention proportions. Subsequently, as the intervention proportion continues to rise, the disease spread is significantly controlled, which in turn allows the epidemic threshold to increase back up. As a whole, while a low intervention proportion can induce a lower infection density, the reduction of the epidemic threshold still leaves the population potentially at risk of infection. Echoing the conclusions just drawn, while a too-low intervention proportion saves public resources, it does not provide a complete preventive effect and is also not an optimal response strategy during an outbreak.

Overall, we suggest that the results under an optimal intervention proportion should exhibit lower infection density and higher epidemic threshold compared with the no-intervention scenario. Through observing the simulation results at a full range of intervention proportions, we have summarized the optimal intervals that meet our requirements, which are labelled in grey in figure 5. We recommend adopting the intervention proportion within this interval, as it ensures that both infection density and epidemic thresholds are in a better state and avoids the waste of resources.

Moreover, from equation (2.6), it can be found that the government response strength d is the behavioural parameter that has the strongest effect on the epidemic threshold. This is also in line with the results revealed by the sensitivity analysis in [38]. Therefore, we observe the impact caused by the variation of d in figure 5. It can be found that in terms of the optimal intervention proportion interval, the ego-based strategy is more sensitive to changes in the strength of government response. When the response strength decreases, the optimal interval shrinks, which should be paid more attention to in the process of practical application. It is important to note that both the response strength and the intervention proportion are essentially determined by government departments. We thus call on policy-makers to combine multiple management ideas and find ways at a broader level of implementing effective disease control strategies that conserve public resources.

## 5. Real-world network simulations

Up to this point, we have achieved the goal of impeding disease spread through the proposed behavioural modulations. In fact, the contact networks of individuals in the real world exhibit more complex characteristics. To verify the usability of the behavioural modulation means in realistic situations, we choose the following two representative network data, denoted as SG and HT09. The former represents the contact network formed by visitors to an art-science exhibition held in Dublin, Ireland, and the latter denotes the contact of participants in the Hypertext Conference held in Turin, Italy [40]. Both types of data are collected under the guidance of the research collaboration project called SocioPatterns,^1^ details of which can be found on the website given in Data accessibility. To obtain the data, each individual participating in the activities is asked to wear a radio-frequency identification badge. When two persons are close enough and face to face, the radio signals in the badges will be exchanged. The time interval between two adjacent radio signal transmissions is set to 20 s. From this, individual contact data are derived at different time steps during the activity, and the final derived contact data will exhibit time-varying properties. Further pre-processing work is performed to make the data more suitable for the constructed behaviour-disease model here. To begin with, individual contact data at different moments are transformed into an adjacency matrix for the population network. Then, we perform an integration process as defined in [41] on the adjacency matrices of different time steps within a day, and the integrated network is used as the actual network for subsequent simulations. The purpose of integrated operations is to generate a network structure reflecting long-term interaction patterns by integrating connections between nodes’ activities within a time window. After integration, the network not only satisfies the static assumption but also accurately reflects key characteristics such as node centrality. We hope to validate the key findings of this paper in real-world scenarios, including the periodicity driven by behavioural factors and the optimal intervention proportion interval for behavioural modulation means.

**Figure 6.**
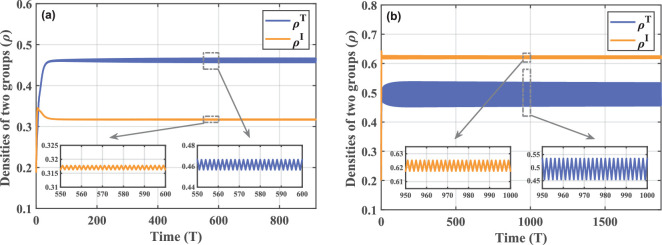
Time series of densities of T-adopters and infected individuals under real-world networks (a) SG, (b) HT09. Parameter conditions are set as $C_{I}=2,C_{T}=0.2,\omega =0.5,d=0.6,\mu =0.5,\beta =0.3$. The number of nodes and time steps are both determined according to the network data. The smaller images embedded in the figures are localized enlargements. The periodic features induced by behavioural factors still persist in real-world situations.

Figure 6 illustrates the evolutionary process of the game–epidemic system 5 under two real networks when no intervention is imposed. The time-series plots reveal that disease spread still behaves in a stable periodic nature, validating the conclusions we obtained previously. It is intuitively noticeable that the disease-spreading process under the two datasets presents different features in several aspects. Examples include the density of T-adopters and those who are infected, amplitude and time required to reach a steady periodic state. Considering that validation efforts need to encompass as wide a range as possible, it would be more appropriate for such differentiated examples to be selected together for simulation. Certainly, such difference also suggests that system dynamics are of crucial relevance to network features.

**Figure 7.**
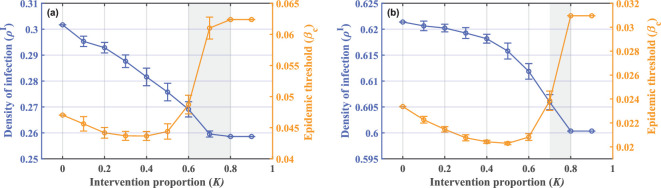
Double curve plot of infection density (left purple axis) and epidemic threshold (right yellow axis) as a function of the number of seed nodes under real-world networks (a) SG, (b) HT09. Parameter conditions are set as $C_{I}=2,C_{T}=0.2,\omega =0.5,d=0.6,\mu =0.5,\beta =0.3$. The number of nodes and time steps are both determined according to the network data. Data points here represent the average of multiple simulations, with error bars based on the standard deviation. It can be observed that simulation results under real-world networks exhibit greater fluctuations, yet the optimal intervention proportion interval we recommend still holds, as indicated by the grey area.

In previous sections, we have found that the ego-based intervention strategy outperforms the alter-based strategy in terms of disease control effects. Hence, representatively, we employ ego-based means in two types of real-world networks to simulate how the epidemic threshold and infection density change with the intervention proportion as shown in figure 7. The emergence of optimal intervention proportion intervals satisfying our proposed criteria under both real networks supports the main conclusions of this study. Just as important is the difference shown in the results under two networks. In figure 7, the trend of epidemic threshold and infection density, and the results for the optimal intervention proportion interval are also influenced by the contact network. Here, we emphasize that during an epidemic, government departments should consider the individual contact patterns in their districts, taking into account the disease control effectiveness and consumption costs, to formulate optimal management plans for controlling epidemic spread.

## 6. Conclusion and discussion

During pandemic events, epidemic courses are critically associated with human behaviour. Interventions that neglect behavioural influences are highly likely to fail to achieve the desired results. For this purpose, we propose a network intervention-based behavioural modulation method to control disease spread. Ego-based and alter-based intervention strategies are applied here to identify seed nodes with high levels of linkage in the network. The strategies of the selected seed nodes are updated to support the government response. As the theoretical foundation for validating the efficacy of proposed behavioural modulation means, a coupled behaviour-disease system on a two-layer multiplexed network is constructed, which simultaneously describes the state evolution on two aspects. Noteworthy, the behaviour layer focuses on the individual’s attitude towards government responses and introduces an evolutionary game framework to portray the strategy transformation mechanism. The time series illustrates that the evolution of decision-making behaviours triggers the periodic feature of the coupled system, highlighting the significant influence of behavioural factors. After applying network interventions, comparative results show that the ego-based strategy performs better in disease control, while the alter-based strategy is more helpful in stabilizing the periodically evolving epidemic situation. More to the point, we identify the optimal intervention proportion intervals that can inhibit disease spread and save management costs, with epidemic thresholds and infection densities as indicators. The above conclusions are validated by simulations under two types of real-world network datasets for accuracy and applicability to real-life scenarios.

In summary, our study opens up a new avenue for efficiently achieving epidemic containment from a behavioural viewpoint. Seed nodes selected through network interventions have the ability to drive more individuals to follow their strategic choices. This enables us to reach control of the entire population by managing the seed nodes on a small scale. The present research can act as a strong reference for government departments in formulating policies on public epidemic management. In practical terms, epidemic control should adhere to the principles of comprehensiveness and flexibility. An optimal approach that takes into account the management costs, response strength, regional characteristics and ultimate outcome will help to overcome the current public health challenges faced by the society. As a final note, some interesting issues are the impact that different two-layer network topologies might have on the behavioural modulation means we propose, and the theoretical foundations of periodic features, which will be left to our future work.

## Data Availability

The real-world network datasets used in this paper are downloaded from the SocioPatterns collaboration, available at: http://www.sociopatterns.org.

## Footnotes

^1^
http://www.sociopatterns.org.

## Appendix A. Notation description

See table 1.

**Table 1.** Definitions of variables and parameters.

notation	description
T,D	strategies of individuals to support (T) or disregard (D) government responses during epidemics
S,I	health status exhibited by individuals as susceptible (S) or infected (I)
β	incidence rate
μ	recovery rate
d	government response strength
ω	selection behaviour intensity
$C_{T},C_{I}$	costs to be paid for supporting the government (T) or being infected (I)
$\pi_{i}^{C}$	pay-off of individual i in state C∈{DS,DI,TS,TI}
$p_{i}^{C}(t)$	probability of individual i to be in state C∈{DS,DI,TS,TI} at time t
$R_{i}^{D}(t),R_{i}^{T}(t)$	probability of individual i in keeping with the current strategy choice D or T unchanged at time t
$q_{i}^{D}(t),q_{i}^{T}(t)$	probability of individual i adopting strategy D or T to remain susceptible at time t

## Appendix B. Supplementary notes for system (2.5)

### B.1. Derivation based on transition probability trees in figure 2

Transition probability trees provide an intuitive representation of transition paths between different states, frequently employed in MMCA modelling research. Each transition probability tree depicts all possible state transition paths that may occur within a single time step, with the root node serving as the initial state. In figure 2, the tree structure comprises two layers that correspond, respectively, to the dynamic processes of the coupled system in terms of behaviour and disease.

Taking the transition probability tree in figure 2a as an example, it starts from the susceptible state that disregards government (DS). We first consider state transitions at the behavioural level. As shown in equation (2.3), the probability of maintaining strategy D unchanged is $R^{D}$, while the probability of updating to strategy T under the influence of other individuals is $1-R^{D}$. Thus, in the first phase, the probability tree generates two branches leading to DS and TS. Next, considering changes in disease states, according to equation (2.4) in the manuscript, the probabilities of remaining susceptible under the two branches are $q^{D}$ and $q^{T}$, respectively. Further, the probabilities of becoming infected can be derived as $1-q^{D}$ and $1-q^{T}$. Thus, in the second stage, a total of four branches is generated, representing the pathways and occurring probabilities of transition from the DS state to DS,DI,TS and TI states. For instance, for individual i, the probability of being in the DS state at time t and remaining in the DS state at time t+1 is denoted as $p_{i}^{DS}(t)R_{i}^{D}(t)q_{i}^{D}(t)$. At this point, we have presented a complete transition probability tree. The construction of the other three trees follows a similar methodology.

Guided by the transition probability tree, the model equations (equation 2.5) under the MMCA framework can be derived. These equations take the probabilities of individual i being in one of four different states at time t+1 as independent variables, describing the relationship between these variables and the individual’s state at the previous time step t. Let us illustrate the derivation process by taking the equation for solving $p_{i}^{DS}(t+1)$ as an example. In a probability tree, the state reached at time t+1 is located at the leaf node position. Therefore, to solve for $p_{i}^{DS}(t+1)$, we need to aggregate all paths starting from the root node in different trees that terminate with the DS state as their leaf node. As illustrated in figure 2, the paths leading to the DS state are DS→DS→DS,DI→DI →DS,TS→DS→DS and TI→DI→DS. By the same token, the paths to the remaining three states are

$$\begin{array}{r} DI:DS\to DS\to DI,DI\to DI\to DI,TS\to DS\to DI,TI\to DI\to DI,\\ TS:DS\to TS\to TS,DI\to TI\to TS,TS\to TS\to TS,TI\to TI\to TS,\\ TI:DS\to TS\to TI,DI\to TI\to TI,TS\to TS\to TI,TI\to TI\to TI. \end{array}$$

Consequently, by summing the transition probabilities corresponding to these paths, we can obtain the MMCA equations in equation (2.5).

### B.2. Simplified cases excluding behavioural factors

In fact, the proposed coupled behaviour–disease system (2.5) in this paper reduces to the standard SIS model after eliminating the influence of behavioural factors. We provide the derivation of this conclusion here as supplementary clarification. First, we set d=1 to ensure that individual decision-making no longer influences disease transmission. Substituting d=1 into equation (2.4) of the manuscript, we obtain

(B1)
$$\begin{array}{lcr} & \begin{array}{rl} q_{i}^{D}(t) & =\prod_{j=1}^{N}\left(1-b_{ji}p_{j}^{I}(t)\beta \right),\\ q_{i}^{T}(t) & =\prod_{j=1}^{N}\left(1-b_{ji}p_{j}^{I}(t)d\beta \right)=\prod_{j=1}^{N}\left(1-b_{ji}p_{j}^{I}(t)\beta \right). \end{array} & \end{array}$$

At this point, the two expressions are equal. Let us denote this as $q_{i}(t)=q_{i}^{D}(t)=q_{i}^{T}(t)$. Furthermore, replacing this expression in equation (2.5) yields the new system as

(B2)
$$\begin{array}{rl} p_{i}^{DS}(t+1) & =p_{i}^{DS}(t)R_{i}^{D}(t)q_{i}(t)+p_{i}^{DI}(t)R_{i}^{D}(t)\mu +p_{i}^{TS}(t)\left(1-R_{i}^{T}(t)\right)q_{i}(t)+p_{i}^{TI}(t)\left(1-R_{i}^{T}(t)\right)\mu ,\\ p_{i}^{DI}(t+1) & =p_{i}^{DS}(t)R_{i}^{D}(t)\left(1-q_{i}(t)\right)+p_{i}^{DI}(t)R_{i}^{D}(t)\left(1-\mu \right)+p_{i}^{TS}(t)\left(1-R_{i}^{T}(t)\right)\left(1-q_{i}(t)\right)\\ & +p_{i}^{TI}(t)\left(1-R_{i}^{T}(t)\right)\left(1-\mu \right),\\ p_{i}^{TS}(t+1) & =p_{i}^{DS}(t)\left(1-R_{i}^{D}(t)\right)q_{i}(t)+p_{i}^{DI}(t)\left(1-R_{i}^{D}(t)\right)\mu +p_{i}^{TS}(t)R_{i}^{T}(t)q_{i}(t)+p_{i}^{TI}(t)R_{i}^{T}(t)\mu ,\\ p_{i}^{TI}(t+1) & =p_{i}^{DS}(t)\left(1-R_{i}^{D}(t)\right)\left(1-q_{i}(t)\right)+p_{i}^{DI}(t)\left(1-R_{i}^{D}(t)\right)\left(1-\mu \right)+p_{i}^{TS}(t)R_{i}^{T}(t)\left(1-q_{i}(t)\right)\\ & +p_{i}^{TI}(t)R_{i}^{T}(t)\left(1-\mu \right). \end{array}$$

Given that $p_{i}^{I}(t)=p_{i}^{DI}(t)+p_{i}^{TI}(t)$, we sum up the corresponding equations for $p_{i}^{DI}(t+1)$ and $p_{i}^{TI}(t+1)$ in the new system resulting in

(B3)
$$\begin{array}{rl} p_{i}^{I}(t+1) & =p_{i}^{DI}(t+1)+p_{i}^{TI}(t+1)\\ & =p_{i}^{DS}(t)\left(1-q_{i}^{,}(t)\right)+p_{i}^{DI}(t)\left(1-\mu \right)+p_{i}^{TS}(t)\left(1-q_{i}^{,}(t)\right)+p_{i}^{TI}(t)\left(1-\mu \right)\\ & =p_{i}^{S}(t)\left(1-q_{i}^{,}(t)\right)+p_{i}^{I}(t)\left(1-\mu \right)\\ & =\left(1-p_{i}^{I}(t)\right)\left(1-q_{i}^{,}(t)\right)+p_{i}^{I}(t)\left(1-\mu \right). \end{array}$$

The above equation exactly satisfies the standard SIS model within the MMCA framework [42].
